# Heritable variation and small RNAs in the progeny of chimeras of *Brassica juncea* and *Brassica oleracea*

**DOI:** 10.1093/jxb/ert266

**Published:** 2013-09-04

**Authors:** Junxing Li, Yan Wang, Langlang Zhang, Bin Liu, Liwen Cao, Zhenyu Qi, Liping Chen

**Affiliations:** ^1^Department of Horticulture, College of Agriculture and Biotechnology, Zhejiang University, Hangzhou 310058, PR China; ^2^Key Laboratory of Horticultural Plants Growth, Development and Biotechnology, Agricultural Ministry of China, Hangzhou 310058, PR China; ^3^Institute of Virology and Biotechnology, Zhejiang Academy of Agricultural Sciences, Hangzhou 310021, PR China; ^4^Agriculture Experiment Station, Zhejiang University, Hangzhou 310058, PR China

**Keywords:** *Brassica juncea*, *Brassica oleracea*, chimera, grafting variation, inheritance, small RNA.

## Abstract

Chimeras have been used to study the transmission of genetic material and the resulting genetic variation. In this study, two chimeras, TCC and TTC (where the origin of the outer, middle, and inner cell layers, respectively, of the shoot apical meristem is designated by a ‘T’ for tuber mustard and ‘C’ for red cabbage), as well as their asexual and sexual progeny, were used to analyse the mechanism and the inheritance of the variation induced by grafting. Asexual TCC progeny were obtained by adventitious shoot regeneration, while TTC sexual progeny were produced by self-crossing. This study observed similar morphological variations in both the asexual and sexual progeny, including changes in leaf shape and the pattern of shoot apical meristem termination. The leaf shape variation was stable, while the rate of shoot apical meristem termination in the TTC progenies decreased from 74.52% to 3.01% after three successive rounds of self-crossing. Specific red cabbage small RNAs were found in the asexually regenerated plants (rTTT) that were not present in TTT, indicating that small RNAs might be transmitted from red cabbage to tuber mustard during grafting. Moreover, in parallel with the variations in phenotype observed in the progeny, some conserved miRNAs were differentially expressed in rTTT and TTT, which correlated with changes in expression of their target genes. These results suggest that the change in small RNA expression induced by grafting may be an important factor for introducing graft-induced genetic variations, providing a basis for further investigating the mechanism of graft-induced genetic variation through epigenetics.

## Introduction

Plant grafting is a well-recognized means of vegetative propagation. It differs from sexual breeding in that it involves germ cells that have undergone meiotic recombination. It is generally thought that grafting does not alter the genetic content of the graft partners (recipient stock/donor scion) or their offspring. However, graft-induced genetic variations (GIGVs) in graft offspring have been well documented in several different experimental systems and plant species (Frankel, 1954; Yagishita, 1961; Ohta and Choung, 1975; Hirata, 1979, 1980a, 1980b; Hirata *et al.*, 1986, 1989; Hirata and Yagishita, 1986; Taller *et al.*, 1998). For example, an earlier study using male sterile petunia for the stock and normal fertile petunia as the scion showed that the male sterility of the stock was transferred to the scion progeny (Frankel, 1954). A later study reported the appearance of genetic variations in mung bean progeny following grafting of the mung bean seedling onto the stem of a sweet potato plant (Zhang *et al.*, 2002). Despite ample experimental evidence for the occurrence of GIGVs, the pattern of inheritance is still unclear, and some data are contradictory. For instance, Ohta (1991) showed that the hereditary changes in certain traits in self-crossed generations of grafted red pepper followed a Mendelian pattern, while Taller *et al.* (1998) described results that did not fit a Mendelian pattern of inheritance. Even though several different mechanisms have been proposed to explain these conflicting results, the nature of GIGVs remains controversial.

Pandey (1976) first proposed that the genetic variation resulted from a transfer of genetic material from the stock to the scion across the graft junction. He hypothesized that DNA fragments transferred from the stock plant integrate into the chromosomes of scion cells during DNA replication by homologous recombination with the scion genome. Later, Zhou *et al.* (2002) attempted to detect evidence of DNA exchange between stock and scion in citrus grafts, but were unsuccessful. Stegemann and Bock (2009) grafted tobacco plants from two transgenic lines carrying different markers and reporter genes in the nucleus and the plastid. They found that the plastid genomes were transferred between plant cells that were in close contact. However, there is no direct evidence that genetic variation can be induced by the transmission of nuclear genomic DNA through grafting.

Grafting between different species involves contact between heterologous cells at the stock and scion junction. It has been previously shown that the cells of the scion and stock species are linked to each other by plasmodesma (PD), and that genetic material can be transported via PD for a short distance (Jackson, 2001). In general, the PD only permits the transfer of some macromolecules, such as specific proteins and RNA (Ding *et al.*, 1999; Zambryski and Crawford, 2000; Oparka and Roberts, 2001; Kim and Pai, 2009). The first endogenous plant protein found to move from cell to cell was KNOTTED1 (KN1), which is a transcription factor that regulates leaf and shoot meristem development (Lucas *et al.*, 1995). Kim *et al.* (2001) reported that the long-distance transport of the mutant Mouse ears (*Me*) transcript correlates with a change in leaf morphology. These results showed that the transmission of proteins and mRNAs from stock to scion can cause changes in phenotypic traits. However, there is no evidence that the variation induced by protein and mRNA transport is inherited and maintained in the offspring. Recently, the biological significance of small RNAs (sRNAs) has attracted much attention. Several groups have shown that endogenous sRNAs can be transmitted during grafting and induce epigenetic modifications such as DNA methylation and RNA silencing, without changing the DNA sequence (Haque *et al.*, 2007; Pant *et al.*, 2008; Dunoyer *et al.*, 2010; Molnar *et al.*, 2010; Wu *et al.*, 2010). However, further research is needed to determine whether sRNAs play a role in the variation induced by grafting.

The periclinal chimera obtained by grafting the shoot apical meristem (SAM) *in vitro* is a special form of grafting (Chen *et al.*, 2006). The SAM of the periclinal chimera consists of three genetically distinct cell layers, named LI, LII, and LIII (from the outer layer to the inner layer). The offspring of a periclinal chimera can be produced from a single layer, by asexual derivation from the LI layer or sexual derivation from the LII layer (Satina, 1945; Zhu *et al.*, 2007). Therefore, the offspring consist of only one genotype, thus enabling the analysis of heritable variation.

This study group previously reported the establishment of several periclinal chimeras by *in vitro* micro-grafting between tuber mustard (*Brassica juncea*) and red cabbage (*Brassica oleracea*) (Chen *et al.*, 2006). The present study further investigates two of these periclinal chimeras, TCC and TTC (where the origin of the outer, middle, and inner cell layers of the SAM is designated by a ‘T’ for tuber mustard or ‘C’ for red cabbage). In these chimeras, cell layers derived from tuber mustard and red cabbage are present in the same SAM. These two species have distinct phenotypic and molecular markers. The goal of this study was to determine the status of sRNAs during grafting and elucidate the origin of GIGVs by analysing variation in the asexual and sexual progeny of single-cell lineages from the chimeras.

## Materials and methods

### Plant materials

Two periclinal chimeras (TCC and TTC) synthesized by *in vitro* grafting between tuber mustard (*Brassica juncea* (L.) Czern. et Coss. var. *tumida* Tsen et Lee.) and red cabbage (*B. oleracea* L. var. *capitata* L.) were used in this study (Chen *et al.*, 2006). The ungrafted tuber mustard and red cabbage plants are referred to as TTT and CCC. Tuber mustard self-grafting was used as a control. All grafts were performed under aseptic conditions.

Explants of chimera nodal segments were cultured on half-strength MS basal medium (Murashige and Skoog, 1962) containing 0.2mg/ml 6-benzyladenine (6-BA) at 25±2 °C under a 16/8 light/dark cycle with 84 μmol m^–2^ s^–1^ fluorescent light. The axillary shoots were excised and transferred to half-strength MS medium for further growth. Chimeric shoots with four or five leaves were then transferred to half-strength MS medium containing 0.1mg/ml α-naphthalene- acetic acid (NAA) for root induction (Zhu *et al.*, 2007). After acclimatization for 10 days in a greenhouse, the rooted plantlets were transplanted to soil. Meanwhile, tuber mustard and red cabbage plantlets were also transplanted to a field for further phenotypic studies.

### Histological analysis and in situ hybridization

The chimeras were identified by histological analysis of the mature leaves (Satina *et al.*, 1940). The mature leaves from chimeras, tuber mustard, and red cabbage were collected for free-hand sectioning. The thinner leaf sections were observed and photographed using an SP 350 Olympus digital camera.

*B. juncea* and *B. oleracea* are AABB and CC genomes, respectively (Maluszynska and Hasterok, 2005). The BB genome is only distantly related to the AA and CC genomes. Genomic DNA from *Brassica nigra* (which has a BB genome) can be used as specific probe to identify *B. juncea* by genome hybridization. The 358bp *atpA* fragment (located in the region from 65,976bp to 66,334bp in *B. oleracea* mitochondrial DNA, but not in *B. juncea*) can be used as a specific probe to identify *B. oleracea*. Thus, these two specific probes were used to discriminate between the T cells and C cells. Genomic DNA was extracted from *B. nigra* and then broken into 200–500bp fragments by boiling in water. The *atpA* fragment was PCR-amplified using the primers *atpAF* (5′-TAAATAAGTAAGACTTGACT-3′) and *atpAR* (5′-GAATTTATAACCCACTAGTC-3′). The two probes were labeled with digoxigenin according to the manufacturer’s instructions (Roche). Hybridization was performed using a modified protocol. The SAMs from the chimeras, tuber mustard, and red cabbage were fixed using Carnoy’s fixative, and paraffin sectioning was performed. The sections were rehydrated, washed with 0.01mol/l PBS for 5min, and incubated with 0.3% Triton X-100 and 5 μg/ml proteinase K for 10min at 37 °C. After treatment with 0.1mol/l glycine-PBS for 5min, the sections were fixed in 4% paraformaldehyde at room temperature. The hybridization mixture consisted of 50% formamide, 4×SSC, 10% dextran sulphate, 5×Denhardt’s, 10% SDS, 0.1 μg/μl herring DNA, and 1 μg/ml labelled probe.

Sections were denatured for 10min at 82 °C, and then exposed to approximately 20 μl of hybridization mixture per section. Hybridization was performed at 42 °C overnight (approximately 18h). The detection was carried out using the DIF High Prime DNA Labeling and Detection Starter Kit І (Roche) according to the manufacturer’s instructions. The hybridized sections were observed and photographed using an Eclipse 90i camera (Nikon). A control hybridization was performed with no probes included in the hybridization mixture, and negative control hybridizations were performed using the *atpA* probe for tuber mustard and the BB genome probe for red cabbage.

### Phenotypic assessment of asexual and sexual chimera progeny

**Fig. 1.**
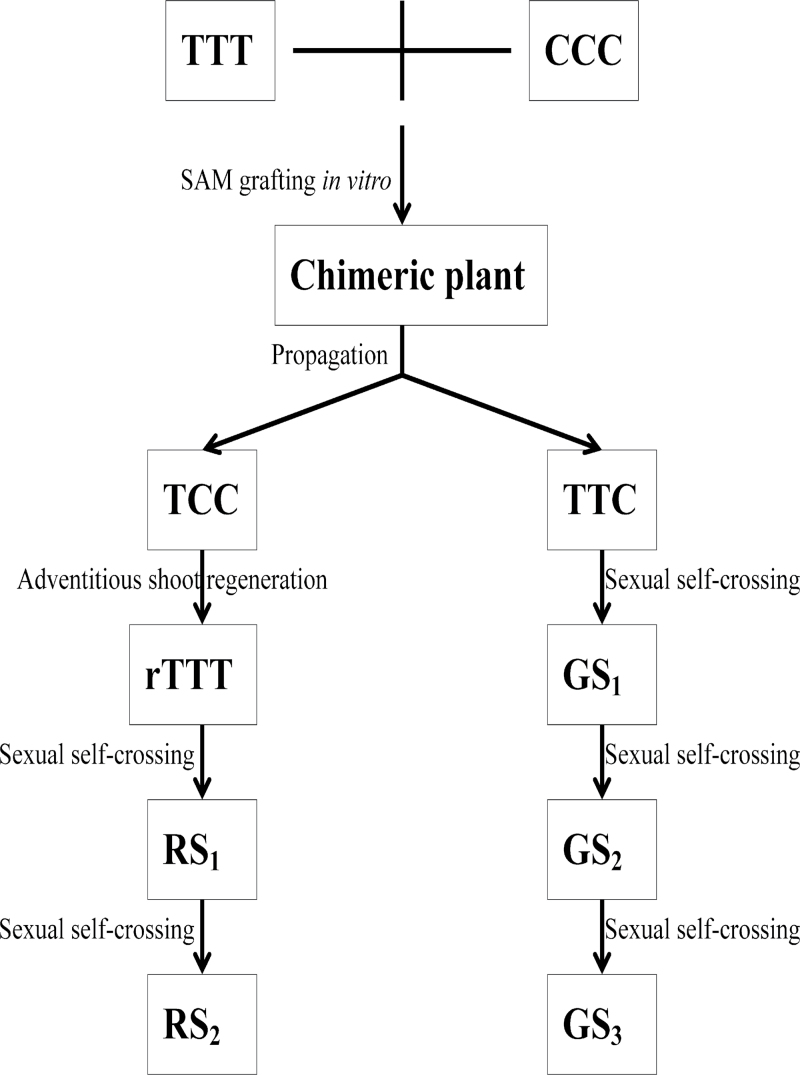
Diagram of grafting and creating the asexual and sexual chimera progenies.

TCC periclinal chimera exhibit complete sterility when used as the female parent (Wang *et al.*, 2011). Asexual TCC progeny were obtained by the regeneration of LI cells under aseptic conditions. First, nodal segments from the TCC chimera were cultured on half-strength MS medium supplemented with 0.2mg/ml BA. Next, the axillary buds were excised and cultured on MS medium supplemented with 3mg/ml BA to induce adventitious shoots. After the adventitious shoots reached 0.5cm in length, they were excised and transferred to half-strength MS basal medium containing 0.2mg/ml 6-BA for further propagation. The new shoots were designated reverted TTT (rTTT), and the first and second generations of rTTT progeny obtained by normal self-crossing were named RS_1_ and RS_2_ (Fig. 1).

TTC periclinal chimera are fertile and can produce sexual progeny by self-crossing. The first self-crossed line derived from TTC was named GS_1_, and the successive generations GS_2_ and GS_3_ were derived from GS_1_ (Fig. 1).

The phenotypes of the asexual, sexual, and control progeny were analysed under the same growth conditions.

### Small RNA analysis

The newly regenerated rTTT plants were used to investigate the status of sRNAs in the T cell lineage after contact with C cell lineage. To rule out contamination of the red cabbage cell lineage during rTTT regeneration and confirm the pure LI origin of the TCC asexual progeny (T cell lineage), the candidate rTTT plants were assayed using PCR amplification of *atpA*, a gene present in the mitochondria of *Brassica* species. The specific primer sequences used to amplify *atpA* were 5′-GCTGCTTACAGGAGTTAGCC-3′ (F) and 5′-GTCCAATCGCTACATAGACA-3′ (R), and the PCR amplification reaction was carried as follows: an initial denaturation step at 94 °C for 5min; 35 cycles of 94 °C for 45 s, 55 °C for 45 s, and 72 °C for 1.5min; followed by one last extension step at 72 °C for 10min. This primer pair amplifies fragments of different lengths from tuber mustard and red cabbage DNA (1050 and 1500bp, respectively), allowing for the detection of potential contamination by the red cabbage lineage (Zhu *et al.*, 2007).

To avoid contamination by viruses, bacterial pathogens, and endophytes, grafting and *in vitro* regeneration of rTTT were performed under aseptic conditions. Total RNA from the newly expanded leaves (the fourth leaf) of the rTTT plants and their parent plants (both grown *in vitro*) was extracted with Trizol (Invitrogen) and used to construct sRNA libraries. Three biological replicates were included for each sample. For each sample, the 18–30 nt sRNA fragments were purified, converted to DNA by reverse-transcription PCR, and ligated sequentially to the 5′ and 3′ adaptors. The reverse-transcription PCR products were then sequenced directly using a Solexa sequencer. After removing the adaptor, filtering the low quality tags and cleaning up the contaminants, the unique sequence reads were counted. All of the sRNAs were mapped to the miRBase (release 16), Genbank/Rfam, Repeats, and Exon/Intron databases. If the results from different databases conflicted with each other, the sRNA annotation was selected from one of the databases in the following priority order: Genbank/Rfam, known miRNA, Repeats, Exon/Intron. Any sRNAs that were not annotated in any database were designated ‘unann sRNA’. After the sequences were aligned, the composition and expression of the sRNAs in each sample was determined. The sRNAs that were specific to rTTT or shared with TTT and CCC were determined by sequencing. Because miRNAs regulate the expression of their target genes to guide morphological alteration of plants as developmental cues (Kim and Pai, 2009), the present study compared the expression level (number of reads) of some conserved miRNAs in TTT and rTTT to estimate their correlation with GIGVs.

### Gene expression quantification by reverse-transcription PCR

The relative expression levels of target genes predicted by different expression miRNAs were analysed in the progeny plants compared to TTT, because similar morphological variations appeared in both the asexual and sexual progeny. The target genes were predicted using psRNA Target (http://plantgrn.noble.org/psRNATarget/). Total RNA was extracted from the young leaves of TTT, RS_1_, GS_1_, GS_2_, and GS_3_ using Trizol (Invitrogen) and converted to cDNA for q-PCR analysis. GS_1_, GS_2_, and GS_3_ were selected on the basis of their SAM termination status. The SAM termination grew weaker from GS_1_ to GS_3_. All amplification reactions were performed with specific primers (Table 1) using 20 μl volumes of SYBR (Takara) and the ABI STEPONE Real-Time PCR system. The 25S ribosomal RNA gene was selected as a reference gene for normalization. Three biological replicates were included for each sample.

**Table 1.** Specific primers for quantitative PCR analysis

Primer name	Sequence (5′–3′)
*SPL9-F*	AGTCGGGTCAGATACCAAGG
*SPL9-R*	TGTTCGATACCAGCCACAGT
*PHV-F*	GATTGGTCTTAACCATAGCC
*PHV-R*	GAAGTGGGAAACTGCATCGA
*PHB-F*	GAGGTGAAGCTGACCCAAAT
*PHB-R*	CAGATATTGGACTGATACTGG
*TCP2-F*	GCCAAAGAGACCAAGGAGAG
*TCP2-R*	GTCTGAACCGGGAAATGAGT
*25SF*	CGGTTCCTCTCGTACTAGGTTGA
25SR	CCGTCGTGAGACAGGTTAGTTTT

## Results

### Identification of periclinal chimeras TCC and TTC

**Fig. 2.**
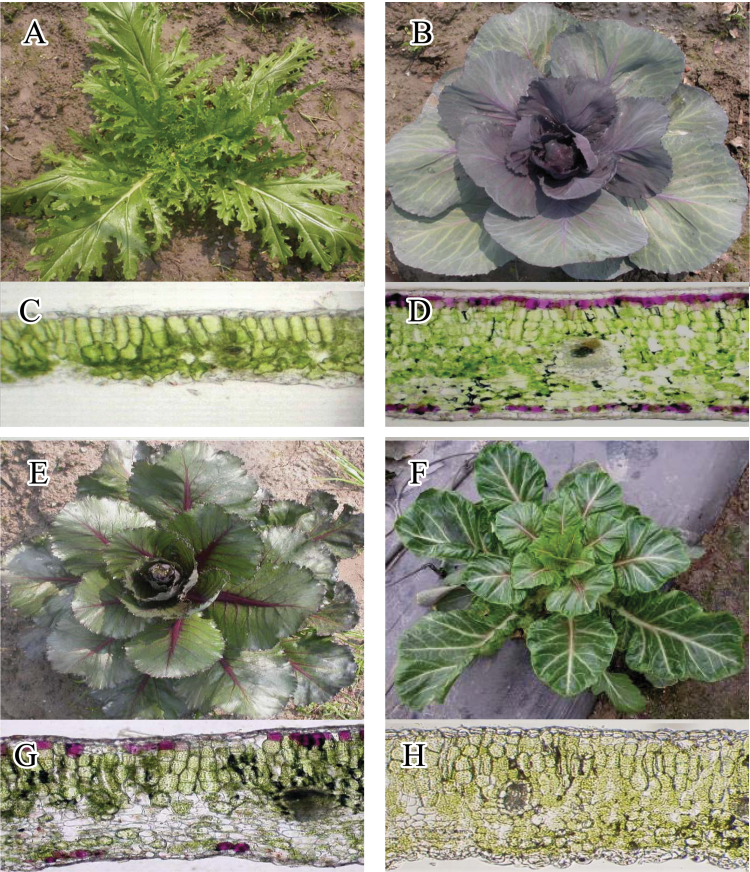
Parent plants and periclinal chimeras: (A) tuber mustard; (B) red cabbage; (C) histological structure of tuber mustard; (D) histological structure of red cabbage; (E) TCC; (F) TTC; (G) histological structure of TCC; and (H) histological structure of TTC. Histological structures taken at magnification ×200 (this figure is available in colour at *JXB* online).

The parental tuber mustard and red cabbage plants have distinct phenotypic markers (Fig. 2A, B). The tuber mustard plant has epidermal hair and green leaves, while the red cabbage plant has smooth, purple, waxy leaves. In addition, tuber mustard leaves are thin, while red cabbage leaves are thick. These morphological characteristics can be used as markers for detecting the two cell lineages. The tuber mustard and red cabbage plants are referred to hereafter as TTT and CCC, respectively.

Two types of chimeras were produced by *in vitro* grafting between tuber mustard and red cabbage (Fig. 2E, F). The chimeras were identified by examining the histological structure of their mature leaves (Fig. 2G, H), using the histological structure of mature leaves from TTT and CCC as controls. As shown in Fig. 2, both chimeras had visible epidermal hairs, suggesting that their LI layer is derived from TTT. The subepidermal layer of one chimera (Fig. 2G) was similar in appearance to CCC (Fig. 2D), indicating that its LII layer is derived from CCC. The subepidermal layer of the other chimera (Fig. 2H) was green (Fig. 2C), indicating that the LII layer originates from TTT. In both chimeras, the cellular morphology and distribution were consistent with those of CCC, with the sponge tissue composed of four or five layers of closely spaced cells, indicating that their LIII layers are derived from CCC. Based on these observations, the two chimeras were named TCC and TTC. Both plants represent periclinal chimeras with a stable phenotype.

**Fig. 3.**
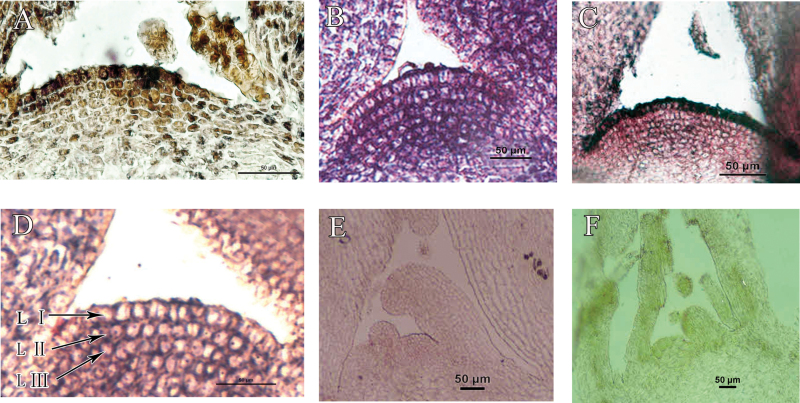
Identification of TCC by *in situ* hybridization. (A) and (B) The tuber mustard and red cabbage SAMs were hybridized with a *B. nigra* genome probe and an *atpA* probe, respectively. (C) Only the LI cells of chimera TCC showed signals when hybridized with the *B. nigra* genome probe. (D) The inner LII and LIII layers hybridized with the *atpA* probe. (E and F) The whole SAM of tuber mustard and red cabbage was hybridized with *atpA* probe and BB genome probe, respectively. LI, LII, and LIII, outmost, middle, and innermost layer of SAM, respectively. Bars, 50 μm (this figure is available in colour at *JXB* online).

The identity of chimera TCC was further confirmed by *in situ* hybridization. As shown in Fig. 3 A and B, the *B. nigra* genome probe and the *atpA* gene fragment probe hybridized with TTT and CCC, respectively, and showed the hybridization signals (black granules in cells). The control samples did not show any hybridization signal (Fig. 3E, F and Supplementary Fig. S1, available at *JXB* online). Using the *B. nigra* genome probe, hybridization signals were observed in the LI (Fig. 3C). Using the *atpA* probe, hybridization signals appeared in the LII and LIII layers (Fig. 3D). These results confirm that the LI layer of the TCC chimera originated from tuber mustard and the LII and LIII layers originated from red cabbage.

### GIGVs in the morphological phenotype of the asexual and sexual progeny of TCC and TTC periclinal chimeras

**Fig. 4.**
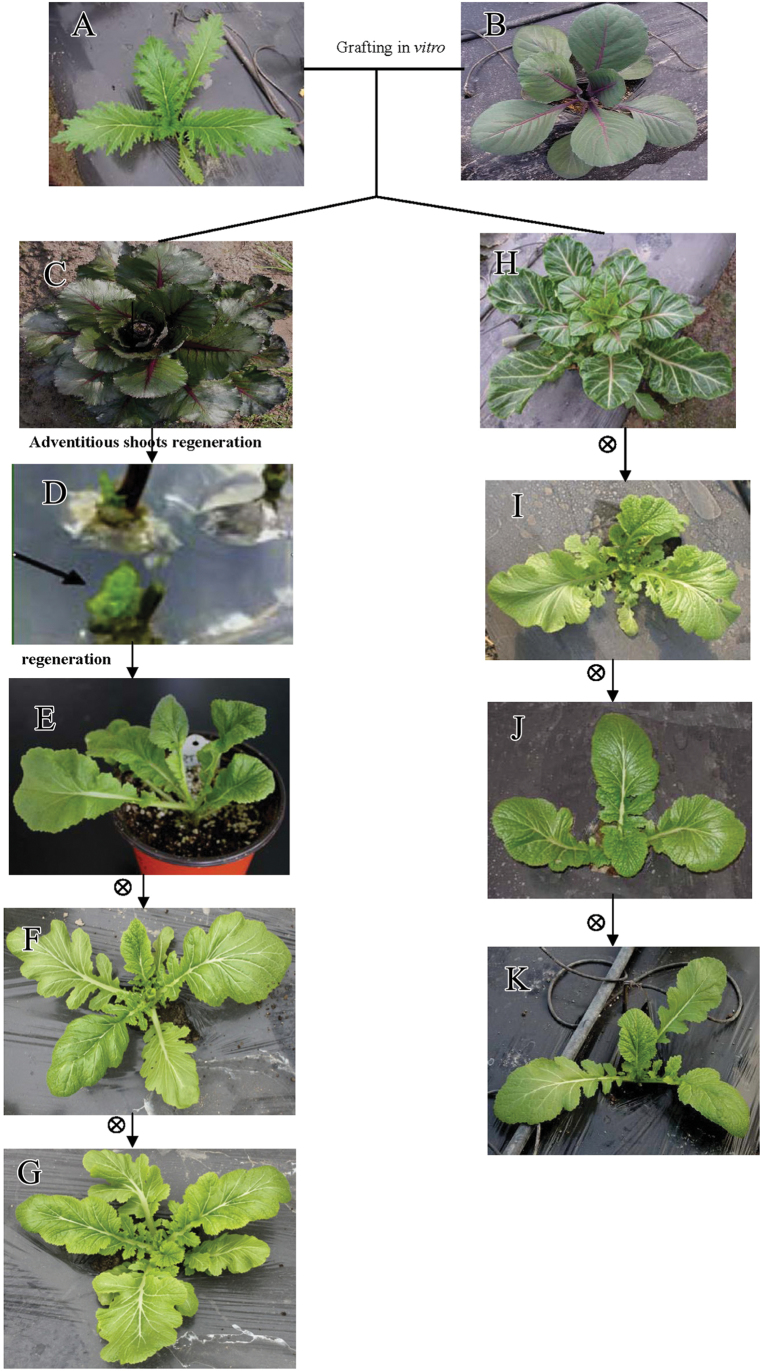
Diagram of grafting and creating the asexual and sexual chimera progenies: (A) TTT; (B) CCC; (C) TCC; (D) adventitious shoots (arrow, indicating self-crossing); (E) rTTT; (F) RS_1_; (G) RS_2_; (H) TTC; (I) GS_1_; (J) GS_2_; (K) GS_3_ (this figure is available in colour at *JXB* online).

**Fig. 5.**
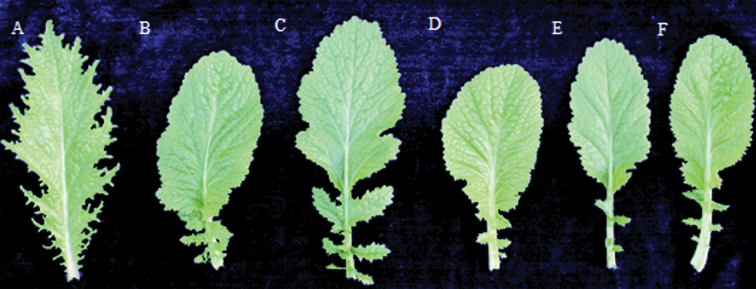
Leaf variation in the chimera progenies. (A) TTT; (B) RS_1_; (C) RS_2_; (D) GS_1_; (E) GS_2_; (F) GS_3_ (this figure is available in colour at *JXB* online).

The phenotypes of the asexual progeny of TCC (rTTT, RS_1_, RS_2_) and the sexual progeny of TTC (GS_1_, GS_2_, GS_3_) were compared to the TTT parent plant (Fig. 4). Phenotypic variations were detected in rTTT, RS_1_, RS_2_, GS_1_, GS_2_, and GS_3_, but not in the progeny of the TTT self-grafted chimera. rTTT, the asexual progeny of TCC, showed remarkable phenotypic variation in leaf shape and SAM growth termination compared to TTT. In particular, the abaxial leaf margins were entire and undulate, resembling the red cabbage to some degree, while the adaxial portions were split to the midrib (Fig. 5). The leaf variation was propagated by self-crossing without segregation (Figs 4 and 5). There was a slight degree of SAM termination in rTTT.

**Fig. 6.**
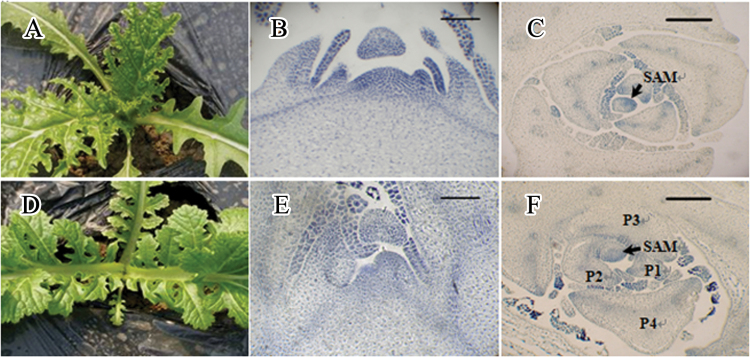
SAM degradation in GS_1_ and histological analysis: (A) TTT; (B) transverse section of TTT SAM; (C) longitudinal section of TTT SAM; (D) GS_1_; (E) transverse section of GS_1_ SAM; (F) longitudinal section of GS_1_ SAM. Bars, 100 μm (this figure is available in colour at *JXB* online).

Interestingly, similar phenotypic variations were observed in the sexual offspring (GS_1_) of the TTC chimera. The variation in leaf shape was propagated to the GS_2_ and GS_3_ progeny (Figs 4 and 5). However, the SAM variation was different in the sexual and asexual progeny. Significant SAM termination was observed in GS_1_ at the fourth-leaf stage (Fig. 6D). As shown in Fig. 6E and F, the dome dispersed to the peripheral primordia, and its size was much smaller compared TTT, as well as being connected with and integrated to the young leaf primordia P1, P2, and P3. Notably, this SAM termination is an inheritable and unstable phenotype. Among the sexual progeny, the frequency of SAM termination progressively decreased in succeeding generations of self-crossing (Table 2).

**Table 2.** SAM termination rate of three self-crossing generations

Sample	Total plantlets	Total terminated	Termination rate (%)
GS_1_	214	160	74.52±3.47
GS_2_	251	29	11.95±2.04
GS_3_	260	8	3.01±1.54

### Analysis of specific small RNAs

**Fig. 7.**
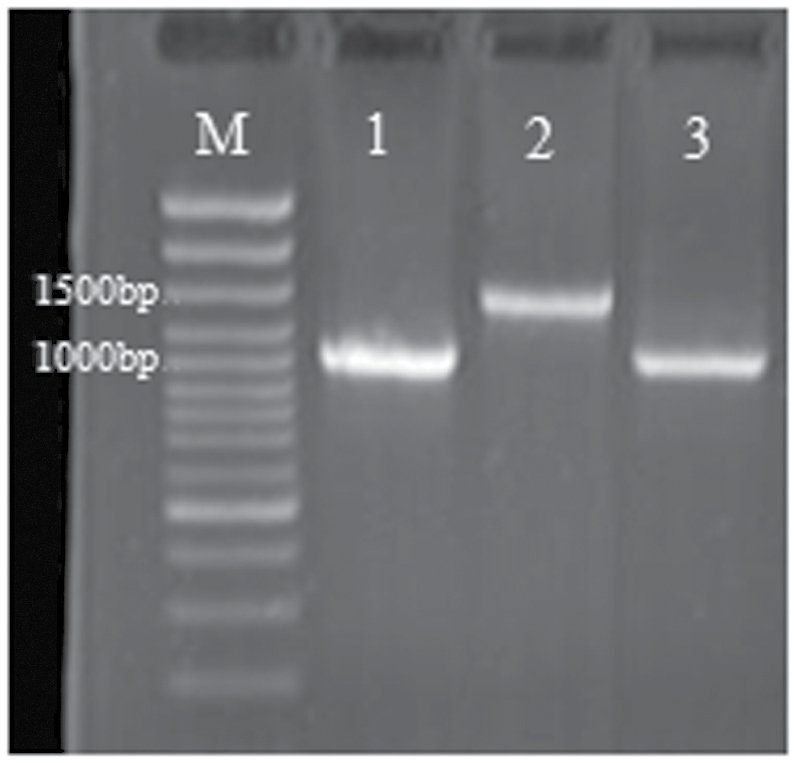
PCR analysis of the *atpA* gene in TTT, CCC, and rTTT. Lanes: M, marker DNA; 1, TTT; 2, CCC; 3: rTTT

**Fig. 8.**
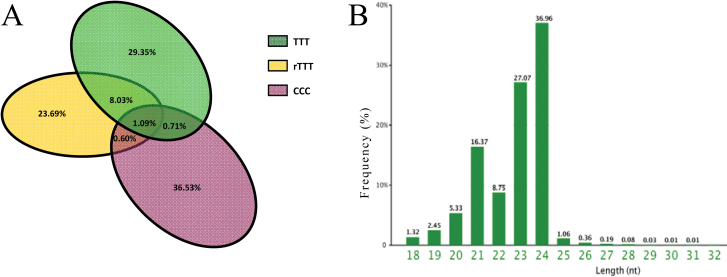
Analysis of unique small RNAs among the three pairwise comparisons (A) and length distributions of the sRNAs transmitted between the two lineages (B) (this figure is available in colour at *JXB* online).

As shown in Fig. 7, the PCR amplification products of *atpA* from rTTT and TTT yielded bands of the same size (Fig. 7), confirming that rTTT did not contain any cells derived from CCC. To identify any change in sRNAs due to grafting, sRNAs from three samples were sequenced. Overall, 24.29% of the sRNAs expressed by rTTT differed from TTT, and 0.60% overlapped with CCC (the complete results are shown in Fig. 8A). The lengths of the 97,324 sRNAs expressed by rTTT but not TTT were analysed (Fig. 8B). sRNAs that were 23 and 24 nt in length accounted for 27.07% and 36.96% of the total sRNAs, respectively. However, many of these sRNAs are not annotated in databases of known sRNAs. Out of the pool of sequenced sRNAs, only 4846 siRNAs, 399 snRNAs, 37 miRNAs, and 27 snoRNAs have been annotated, and most of them had relatively low reads. The details of the sRNAs (excluding the rRNA and tRNA) with higher abundance (reads ≥20) and miRNAs (with a low abundance) are shown in Supplementary Table S1, although the identity of the majority of these sequences is unknown. Most of the miRNAs were conserved among the TTT, CCC, and rTTT plants. It is presumed that this phenomenon also occurred in the TTC progeny, because the T cell lineage present in the LII layer is in direct contact with the C cell lineage in the LIII layer.

### Expression of conserved miRNAs and their predicted target genes

**Fig. 9.**
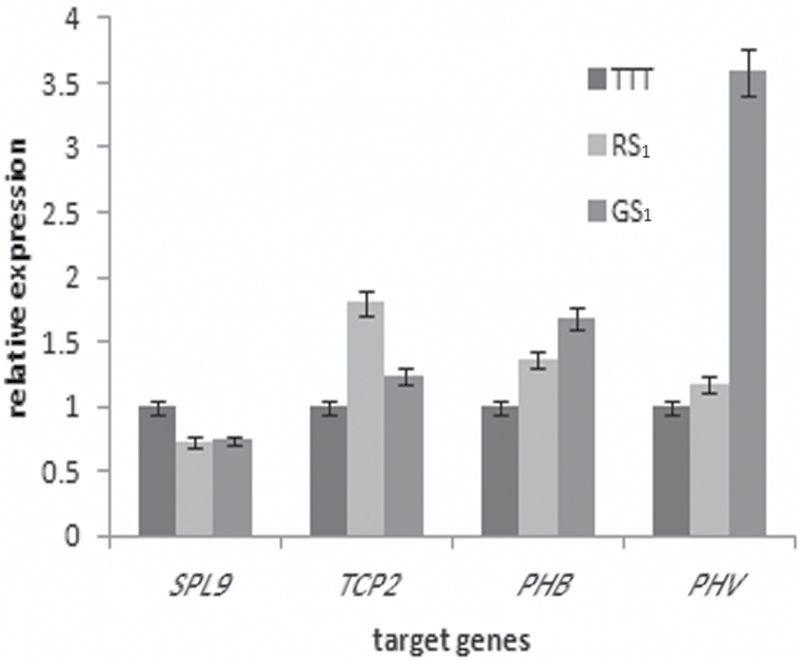
Expression of target genes in TTT, RS_1_, and GS_1_. *TCP2* and *SPL9* are related to the formation of the leaf profile, and *PHB* and *PHV* are related to SAM development.

**Fig. 10.**
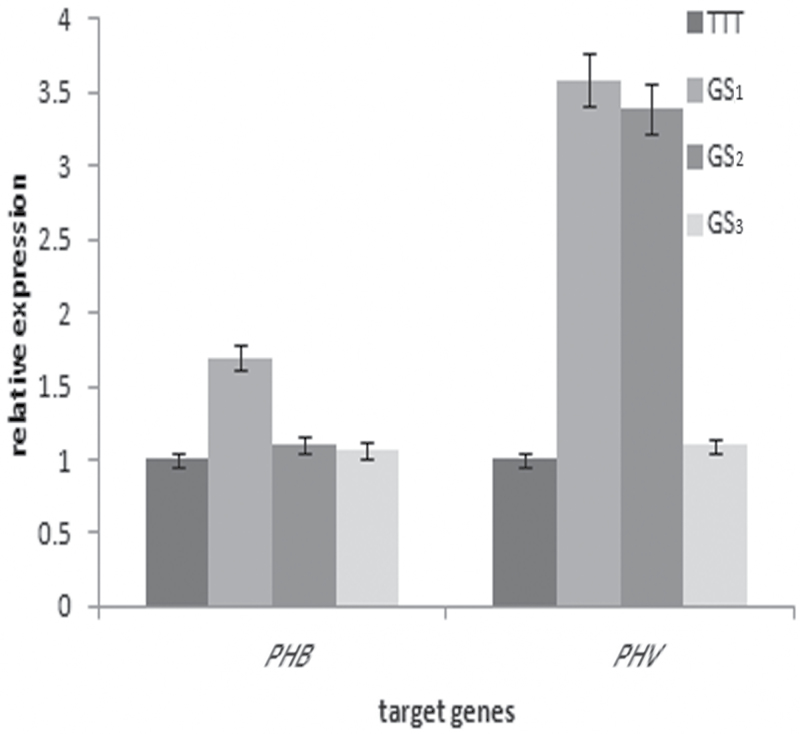
Quantitative PCR analysis of predicted target genes in TTT and TTC sexual progenies: GS_1_, GS_2_, and GS_3_ exhibited a decreasing degree of SAM growth termination.

This study next compared the expression levels of conserved miRNAs in TTT and rTTT whose biological functions are associated with the leaf development and morphology (Table 3). The expression of miR166 and miR319 were all downregulated in rTTT. Their target genes were predicted by combining the phenotypic changes induced by grafting and the genes related to the formation of leaf profile (*TCP2* and *SPL9*) and SAM development (*PHB* and *PHV*) (Williams *et al.*, 2005; Kim *et al.*, 2005; Wu *et al.*, 2009; Pulido and Laufs, 2010). Based on the results observed in rTTT, the predicted target genes were also analysed in the sexual progeny (GS). The expression levels of the target genes in both GS_1_ and RS_1_ were higher than in TTT, except that of *SPL9* (targeted by miR156a) (Fig. 9). In addition, the expression of genes related to SAM changed according to the SAM growth termination status (Fig. 10). In conclusion, changes in the expression level of target genes correlated with changes in the expression level of the corresponding miRNAs.

**Table 3.** Comparison of the conserved miRNAs in rTTT and TTT and their predicted target genes

miR-name	Sequence (5′–3′)	TTT (reads)	rTTT (reads)	Target genes
miR156	UGACAGAAGAGAGUGAGCAC	863,018	1,237,177	*SPL9*
miR166	UCGGACCAGGCUUCAUUCCCC	249,920	189,755	*PHV*, *PHB*
miR319	UUGGACUGAAGGGAGCUCCCU	83	10	*TCP2*

## Discussion

In agriculture and horticulture, grafting is widely used to improve plant vigour, modify plant structure, and increase plant tolerance to stresses or diseases. In general, genetic changes in the graft offspring are not desirable, however, GIGVs often appear. It is therefore important to understand how GIGVs occurs and is passed on to offspring.

### Periclinal chimeras are a suitable system for analysing genetic material exchange during grafting

The SAM of the periclinal chimera consists of genetically different layers, and its composition is traditionally determined by the histological structure of the mature leaves (Satina *et al.*, 1940). This study used *in situ* hybridization to confirm the genetic constitution of the SAM (Fig. 3). Using the *B. nigra* genome probe, hybridization signals were observed in the LI layer (Fig. 3C). Using the *atpA* probe, hybridization signals appeared in the LII and LIII layers (Fig. 3D). This is the first time that SAM composition has been determined at the molecular level. The results showed that the histological analysis was consistent with the molecular analysis. This work then proceeded to use the two chimeras TCC and TTC, in which the SAM composition had been confirmed by histological or molecular analysis, to assess the exchange of genetic material and its effect on succeeding generations.

The periclinal chimera offers specific advantages for studying the transmission of genetic material between heterologous cells. First, the cells in the genetically different layers are adjacent to each other and therefore in more direct contact than in scion-stock grafting (Chen *et al.*, 2006), providing a greater chance for the exchange of genetic material between the different layers. Secondly, the heterologous cells in the SAM of a periclinal chimera can be reseparated and purified through asexual and sexual propagation. For example, the asexual progeny (rTTT) of a single cell lineage (T) can be generated from the LI layer (T) of TCC, which is in tight contact with the LII layer (C). In addition, the sexual progeny (GS) of a single cell genotype (T) can be produced by self-crossing TTC, because all gametes are derived from the LII layer (T) (Satina, 1945), which is in tight contact with the LIII layer (C). The progeny of chimeras derived from the T layer can be used to analyse the exchange of genetic material between the T and C layers. Consequently, studying the genetic characteristics of the asexual and sexual progeny may yield insights into the origin of GIGVs.

### Small RNAs are transmitted between the cells of the graft parents

It is generally believed that the exchange of genetic material between heterologous cells is the key factor for GIGVs. To determine whether sRNAs are transmitted between heterologous cells across the grafted SAM conjunction, this study analysed plants regenerated from the single cell lineage of a periclinal chimera by asexual method, since sRNA would be degraded or diluted by sexual propagation. The TCC chimera was used to determine the status of sRNAs in the T cell lineage after contact with the C cell lineage. The plant samples were obtained under aseptic conditions to eliminate contamination by genetic material from other organisms. Before sequencing the sRNAs isolated from rTTT, PCR analysis ruled out any possible contamination from red cabbage cells (Fig. 7).

In previous studies, sRNA transmission has been analysed in stock–scion grafts. Analysis of the sRNAs in rTTT revealed a change in the specific sRNAs expressed by the T cell lineage after contact with the C cell lineage (Fig. 8A). Specific sRNAs detected in rTTT were expressed in CCC but not in TTT, suggesting that there may be an exchange of sRNAs between cells of the T and C layers. There was a wide range in the expression level of these sRNAs, from less than 10 reads to 100 reads, suggesting that sRNAs with different biological functions are differentially regulated. Thus, these data may be a starting point for future studies investigating the transmission of heterologous sRNAs.

Although this work did not investigate the transmission of sRNAs in the sexual progeny derived from the LII layer of TTC, it is presumed that sRNAs from the C lineage can be transmitted the T cell lineage too, due to the contact of T and C cell lineages in the LII and LIII layers. Further research is needed to determine whether the effect of sRNA transmission on TCC asexual progeny is consistent with the effect in TTC sexual progeny.

### GIGVs may be caused by changes in small RNAs and inherited genetically and epigenetically

As described above, plantlets of the T cell lineage segregated from chimeric tissues by asexual and sexual propagation exhibited similar variations after a cell-contact period with cells of the C lineage. GS_1_ and rTTT plants were homozygotes, as no further segregation of leaf morphological traits was observed in their self-crossed progenies. This conclusion is consistent with a previous study in which the variation induced by grafting changed from a dominant homozygous to a recessive homozygous trait (i.e., both alleles changed at the same time) (Ohta, 1991). The variation in leaf morphology was reproducible and directional, suggesting that it was guided by red cabbage cells by an undefined mechanism. However, the heritable pattern of the SAM growth termination was different from that of leaf morphology, as it was unstable and exhibited occasional reversions in the progeny. Therefore, the SAM and leaf morphology variation have independent patterns of inheritance.

It was previously reported that sRNAs from one parent plant can regulate found novel targets in the genome or transcriptome of the opposite parent plant, and that 24-nt sRNAs are associated with the methylation of DNA with similar sequences (Hamilton *et al.*, 2002; Baulcombe, 2004; Chan *et al.*, 2004). Moreover, epigenetic states can be inherited by sexual progeny in a Mendelian manner (Kakutani, 2002; Zilberman and Henikoff, 2005). In the present study, the 23- and 24-nt sRNAs accounted for a significant proportion of the specific sequence sRNAs (Fig. 8B). Because leaf variations were observed in successive generations, it could be that epigenetic modifications or other changes induced by the transmitted sRNAs play an important role in leaf variation. However, due to the insufficiency of siRNA databases and genomic information for tuber mustard, specific epigenetic changes could not be identified. However, changes in epigenetic states have been observed by other groups. For example, Jacobsen and Meyerowitz (1997) found an epigenetically inherited allele of the *SUPERMAN* (*SUP*) gene could revert to the normal allele. The present study observed a similar phenomenon for SAM growth termination. The frequency of SAM termination progressively decreased in the sexual progeny and it is hypothesized that the methylation pattern of genes related to SAM development may have reverted to the original state after several generations. This study group is currently investigating DNA methylation to understand how the heterologous siRNAs are exchanged and their roles in the induction of GIGVs.

It has been reported that gene regulation through sequence-specific interactions between miRNAs and their target genes can affect plant growth and development (Zhou et al., 2007; Kim and Pai, 2009). Many mature miRNA sequences are conserved among evolutionarily related plant species. This work found that the miRNAs in rTTT and TTT were well-conserved, although the expression levels of the conserved miRNAs differ (Table 3). The expression of the predicted target genes varied along with quantitative changes in the conserved miRNA and progeny phenotypes. For example, Williams *et al.* (2005) reported that the downregulation of *PHB* and *PHV* miRNA leads to upregulation of *WUS* transcription in the organizing centre of SAM, causing SAM enlargement (Kim *et al.*, 2005). In the present study, the expression levels of *PHB* and *PHV* were higher in the progeny than in TTT, which correlated well with the observations of SAM growth termination. Furthermore, the expression levels of *PHV* and *PHB* differ with different ratios of SAM growth termination in plants. The function of two genes reported in another previous study (Wu *et al.*, 2009; Pulido and Laufs, 2010) supports the present observation of the relationship between expression level and phenotype. Furthermore, the expression of target genes was consistent with the results of digital gene expression profiling in TTT and variation progeny (JX Li, LW Cao, and LP Chen, unpublished results). These findings strongly implicate that grafting can affect the expression level of miRNAs. However, further research is needed to gain a better understanding of how grafting affects miRNA expression levels.

In conclusion, this study has provided a detailed characterization of GIGVs in periclinal chimeras of *Brassica*, as well as conclusive evidence for changes in sRNA during grafting. However, GIGVs induced by the exchange of heterologous sRNAs may only occur under certain conditions. This study proposes that the diverse patterns of GIGVs are due not only to the transmission of heterologous sRNAs but also to genetic and epigenetic changes in novel targets of the heterologous sRNAs in the recipient cells.

## Supplementary material

Supplementary data are available at *JXB* online.

Supplementary Table S1. The identified sequences of sRNAs transmitted between the TTT and CCC lineages.

Supplementary Fig. S1. Negative control hybridizations.

Supplementary Data
