# Supplementary material for: Heritable variation and small RNAs in the progeny of chimeras of Brassica juncea and Brassica oleracea
Source: J Exp Bot. 2013 Sep 4;64(16):4851–62. doi: 10.1093/jxb/ert266 (PMC3830474; doi:10.1093/jxb/ert266)
Supplement: Supplementary Data [file supp_64_16_4851__index.html]

Heritable variation and small RNAs in the progeny of chimeras of Brassica juncea and Brassica oleracea — Heritable variation and small RNAs in the progeny of chimeras of Brassica juncea and Brassica oleracea — Supplementary Data 

# Heritable variation and small RNAs in the progeny of chimeras of *Brassica juncea* and *Brassica oleracea*

## Supplementary Data

Data files

**Files in this Data Supplement:**

- Supplementary Data - Supplementary Data
