# Supplementary material for: Heritable variation and small RNAs in the progeny of chimeras of Brassica juncea and Brassica oleracea
Source: J Exp Bot. 2013 Sep 4;64(16):4851–62. doi: 10.1093/jxb/ert266 (PMC3830474; doi:10.1093/jxb/ert266)
Supplement: Supplementary Data [file supp_ert266_jexbot097725_file001.pdf]

Heritable variations and small RNAs in the progeny of the chimeras between *Brassica juncea* and *Brassica oleracea*

Junxing Li, Yan Wang, Langlang Zhang, Bin Liu, Liwen Cao, Zhenyu Qi, and Liping Chen

Supplementary Data

Table S1 The identified sequences of sRNAs transmitted between the TTT and CCC lineages

**Sequences of sRNA（5'→3'）**	**Type**	**TTT**	**rTTT (Code-reads)**	**CCC (Code-reads)**
AAGGTACATGTGGAATGCGGTTA	unann	-----	t0000960-538	t0002237-424
AAAGGTACATGTGGAATGCGGTTG	unann	-----	t0001593-356	t0033213-37
AAAGGTACATGTGGAATGCGGTT	unann	-----	t0004339-157	t0021041-57
ATGCTGAGAACTGATAGCAAGC	unann	-----	t0005144-136	t2420289-1
TATGCTGAGAACTGATAGCAAGCT	unann	-----	t0006260-116	t5483730-1
AAGGTACATGTGGAATGCGGTTGC	unann	-----	t0006485-112	t0124097-11
ATGCTGAGAACTGATAGCAA	unann	-----	t0007053-104	t0740978-2
GTAAAGGTACATGTGGAATGCGGT	siRNA	-----	t0008369-90	t0064305-20
AAGGTACATGTGGAATGCGGTTG	unann	-----	t0009102-84	t0025873-47
TTTGGCTCTGTTTCCTTCTCT	unann	-----	t0010490-74	t0071813-18
TACATGTGGAATGCGGTTGCCTA	unann	-----	t0014780-56	t0144381-10
GTAAGAACTCAGGAAGAAGAACGA	unann	-----	t0014890-55	t0000963-882
TGTAAAGGTACATGTGGAATGCGG	unann	-----	t0015927-52	t0077017-17
GCGGAAGATGTAACGGGGCTAAGCAG	unann	-----	t0017282-49	t0118751-12
AGTGAAATCGAGTAGGACGGA	unann	-----	t0017809-47	t2650510-1
CTCTATGCTGAGAACTGATAGCA	unann	-----	t0019905-43	t1118869-2
GTGCAATCTCGGGAGACAGACATC	unann	-----	t0020635-42	t0027997-44
ATACACGGCGTTAGATTTATATAA	unann	-----	t0020165-42	t0784427-2
AAAAGTAGTAACCATACAAGTCCA	unann	-----	t0022027-39	t0041261-30
ACAGACATCAGAGACAAGGTTAAT	unann	-----	t0022843-38	t0009445-119
TATGCTGAGAACTGATAGCAA	unann	-----	t0023115-38	t5178547-1
AAGTGAAATCGAGTAGGACGGA	unann	-----	t0026741-33	t0957925-2
GGGGGACGGACTGGGAACGGCTCTTTC	unann	-----	t0027657-32	t0009118-123
AGGAGAATAGACGATGATTGGTAT	unann	-----	t0028004-32	t0062501-21
ATCGGTTACTCTGTTTCAGAAGAT	siRNA	-----	t0027573-32	t0102285-13
AGTGAAATCGAGTAGGACGG	unann	-----	t0027394-32	t1964653-1
ACTGGACCAAGAAGTATTAGGGCT	unann	-----	t0029138-31	t1290252-2
ACGTGGCAACGGTAGTGTGAATA	unann	-----	t0028814-31	t4280190-1
ATGTGGAATGCGGTTGCCTAGATA	unann	-----	t0030271-30	t5747995-1
GGACGGACTGGGAACGGCTCTT	unann	-----	t0032365-28	t0002627-372
AAGATGTAACGGGGCTCAAGCGGT	unann	-----	t0032523-28	t0609299-3
GCAATCTCGGGAGACAGACATC	unann	-----	t0034661-26	t0057436-22
GCTGAGAACTGATAGCAAGCT	unann	-----	t0036719-25	t3200630-1
AAAGGTACATGTGGAATGCGGTTA	unann	-----	t0038979-24	t0127361-11
TTAAGTGGGAAACGATGTG	unann	-----	t0039511-24	t0688133-3
AATCTCGGGAGACAGACATC	unann	-----	t0038576-24	t1005291-2
CGGGGGACGGACTGGGAACGGCTCTTT	unann	-----	t0041281-23	t0013657-85
TCGTGTCCCGGCGACGGAGCCA	unann	-----	t0041164-23	t5423306-1

AGTAACTCAGCTGAATTGTGGACA	unann	-----	t0042093-22	t0025670-47
TTAATGCCGAGAACTGATGACGATCCT	unann	-----	t0041578-22	t1399293-1
TTTAGGTAGCGCCTCGGACG	unann	-----	t0045590-21	t5219285-1
AAAGACAAACAGAGCGACAAAGAA	unann	-----	t0046669-20	t0032140-38
AGGTACATGTGGAATGCGGTTA	unann	-----	t0046916-20	t0046444-27
CAATCTCGGGAGACAGACATC	unann	-----	t0046674-20	t0060669-21
AACAGACCGGCAAACACTCAGCAT	unann	-----	t0046729-20	t0171980-8
ATGTAGCGACTGGTGTATGATTTC	unann	-----	t0048460-20	t0439762-4
GAGACTAGAACTCATAAGGAAGAA	unann	-----	t0046996-20	t0899181-2
AAGTGAAATCGAGTAGGACGG	unann	-----	t0047021-20	t2981912-1
TGAAGCGGTGGAGGCGTCTGTTA	unann	-----	t0046366-20	t1792853-1
ATCATGCGATCTCTTTGGATT	miRNA	-----	t0143145-8	t4900520-1
AGTAGCCAAGGATGACTTGCCTG	miRNA	-----	t0241371-5	t0095499-14
TTGACAGAAGATAGAGAGCACAGA	miRNA	-----	t0247812-5	t4164689-1
GCTCACTGCTCTTTCTGTCAGAT	miRNA	-----	t0369298-4	t0339552-5
GACAGAAGAAAGAGAGCAC	miRNA	-----	tt0827890-2	t3583171-1
TCAATAAAGCTGTGGGAAGATA	miRNA	-----	t0856757-2	t3827040-1
TGATTGAGCCGTGTCAATATC	miRNA	-----	t1797662-1	t0790631-2
TGACAGAAGAAAGAGAGCACA	miRNA	-----	t3631105-1	t0043530-29
GGTGACAGAAGAGAGTGAGCA	miRNA	-----	t4425956-1	t1203125-2

-----stands for sRNAs not observed in TTT

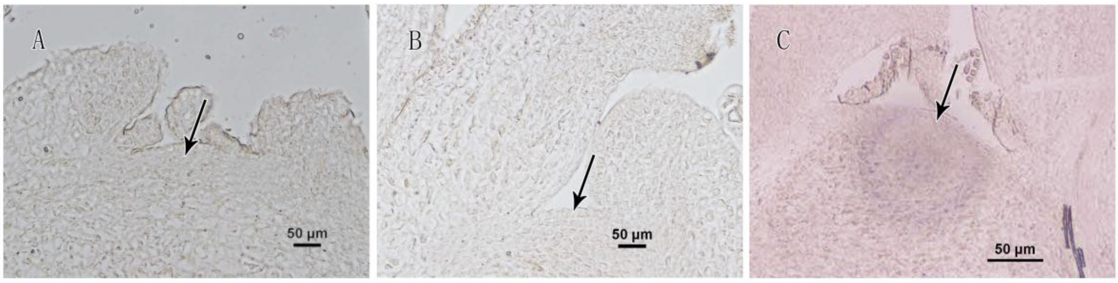

Fig. S1 The control hybridization. A, B and C: The whole SAM of tuber mustard, red cabbage and one chimera was respectively hybridized without containing probes in hybridization mixture. Bar=50μm, the arrows indicate the SAM.
